# Key operational challenges at a programmatic level to achieve optimal use of the GeneXpert platform

**DOI:** 10.5588/ijtldopen.25.0630

**Published:** 2026-05-11

**Authors:** I. Cuella-Martin, F. Hakizayezu, E. Kamanzi, Y.M. Habimana, P. Migambi, B.C. de Jong, L. Rigouts, J.C.S. Ngabonziza

**Affiliations:** 1 Mycobacteriology Unit, Department of Biomedical Sciences, Institute of Tropical Medicine, Antwerp, Belgium;; 2 Department of Biomedical Sciences, Antwerp University, Antwerp, Belgium;; 3 National Reference Laboratory Division, Department of Biomedical Services, Rwanda Biomedical Centre, Kigali, Rwanda;; 4 Tuberculosis and Other Respiratory Diseases Division, Rwanda Biomedical Centre, Kigali, Rwanda;; 5 Research Innovation and Data Science Division, Rwanda Biomedical Centre, Kigali, Rwanda;; 6 Department of Clinical Biology, University of Rwanda, Kigali, Rwanda.

**Keywords:** tuberculosis, TB diagnosis, mWRD, molecular diagnostic, Rwanda, laboratory workflow

## Abstract

**BACKGROUND:**

Although Xpert MTB/RIF Ultra (Ultra) has revolutionised TB diagnosis, operational challenges can significantly undermine its benefits. This study examined operational experiences and challenges associated with Ultra use across Rwanda’s health care settings.

**METHODS:**

We conducted a cross-sectional survey across 53 GeneXpert testing centres between October and December 2024. The survey addressed machine usage, sample processing timelines, and technical challenges through structured interviews with laboratory managers and operators.

**RESULTS:**

While 41% of sites processed samples within 5 h, 55% experienced delays exceeding 24 h due to high volumes and limited capacity. Four labs (8%) reported significant delays (>8 h) processing sputum with sample reagent. Modules failures occurred in 81% of facilities within the preceding year, with most experiencing infrequent failures and one site experiencing more than six annually. Connectivity issues with internet and/or ‘DataToCare’ affected 17% of sites, causing workflow delays. Equipment capacity constraints were identified in 30% of sites, particularly affecting facilities managing dual TB and HIV/hepatitis testing, while 9% reported staffing shortages.

**CONCLUSION:**

Despite successful nationwide deployment, operational challenges limit Ultra’s full potential. Strategic machine allocation, upgraded connectivity, and improved preventive maintenance represent key intervention areas. These findings offer valuable insights optimising rapid molecular diagnostic use in similar settings.

The GeneXpert platform, a molecular WHO-recommended rapid diagnostic (mWRD), combined with the Xpert MTB/RIF assay, has revolutionised TB diagnosis in resource-limited settings by offering rapid molecular testing with significantly enhanced sensitivity and specificity compared to conventional microscopy.^1^ The development of the newer Xpert MTB/RIF Ultra (Ultra) assay has further enhanced sensitivity, particularly among patients with paucibacillary TB.^2^ Although performing the GeneXpert assay is relatively simple with minimal manipulation of the specimen, operational challenges such as sample processing delays, device malfunctioning, training gaps, and ineffective supply chain can significantly undermine the benefits of the GeneXpert diagnostic platform.^3^ The manufacturer recommends an ambient operating temperature for the instrument between 15°C and 30°C, with modules requiring annual calibration.^4^ Previous research on GeneXpert deployment in resource-constrained settings has documented that unless systems are implemented with comprehensive supporting tools (such as trained staff, reliable power supply, sample transport network, and integrated data systems) and within the context of a strengthened health system, diagnostic tests alone are unlikely to produce the expected impact on the TB care cascade.^5,6^ Systematic approaches to diagnostic network optimisation (DNO) have emerged as valuable tools for evidence-based decision-making around strategic placement of diagnostics and design of optimal sample referral systems to balance patient access with operational efficiency.^7^

In 2022, Rwanda’s National Tuberculosis Program (NTP) transitioned gradually from the classic Xpert MTB/RIF to Ultra.^8,9^ During the 2023–2024 fiscal year, 107,825 GeneXpert tests were performed for initial diagnostics among patients with presumptive TB, and for initial rifampicin-susceptibility testing of smear-positive TB patients, reaching a 52% utilisation rate for TB testing.^10^ External quality control demonstrated strong performance, with 97% (66 of 68) of GeneXpert sites receiving proficiency panels from the Centers for Disease Control and Prevention (Atlanta, USA). Among the 66 laboratories enrolled, 94% (62/66) achieved passing marks of 80% and above, while 6% (4/66) failed to meet the quality standards. Understanding how laboratory workload and performance levels relate to operational challenges can help target quality improvement interventions. During the same year, Rwanda expanded its network of GeneXpert machines from 70 to 91 sites, aiming to further improve rapid TB diagnosis and surveillance for rifampicin-resistant TB.^10^ To maximise the utility of this investment, additional testing capabilities have been integrated into the GeneXpert platform, including early infant HIV diagnostics and hepatitis-C viral load testing.^10^ To improve connectivity and data management, Rwanda introduced DataToCare open-source software in 2018 and upgraded it in August 2022 as a customised application intended to support the TB division’s data collection needs. Despite the system upgrade, the implementation of DataToCare has not been optimal, with only 41.4% of MTB-detected results successfully reported through the system in the 2023–2024 fiscal year in Rwanda, due to technical incompatibilities with GeneXpert, issues with Windows versions, and user adoption challenges.^10^

The assessment of GeneXpert users’ experience offers a valuable opportunity to evaluate the operational challenges affecting this advanced diagnostic platform within a coordinated NTP. Understanding these challenges is essential for developing interventions to maximise diagnostic efficiency and, ultimately, improve patient outcomes. This study examined the operational barriers in the use of GeneXpert across Rwanda’s health care settings, with a focus on sample handling procedures, machine functionality, and resource constraints that could limit the technology’s effectiveness.

## METHODS

**Figure 1.**
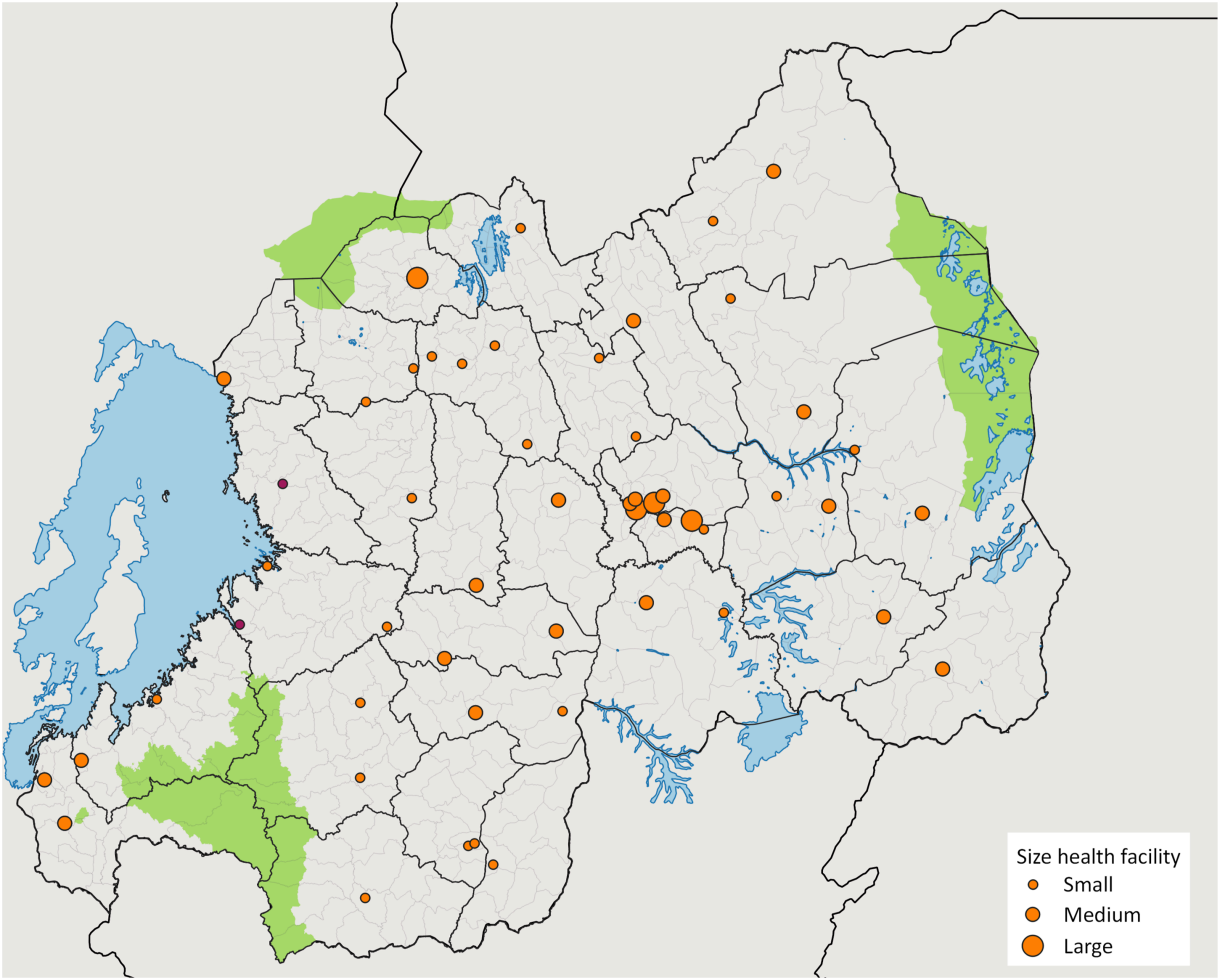
Distribution of the 53 health facilities that participated in the survey. The size of each circle corresponds to the facility’s patient volume, with larger circles representing facilities seeing higher numbers of patients per day. Circles with pink colour indicate facilities where patient volume data were not available.

We conducted a cross-sectional survey across 53 GeneXpert testing centres in Rwanda between October and December 2024. These 53 facilities ensured geographical representation across all provinces and represented 73% of the country’s total testing sites at the time (53/73) (Figure 1). The study population encompassed sites of varying size and daily patient volume, categorised as small (serving <50 patients), medium (51–200 patients), and large (>200 patients). This stratification enabled analysis of operational challenges across different facility types and resource levels.

The survey addressed three categories: 1) GeneXpert machine usage; 2) sample processing and handling procedures, including time intervals at critical processing steps; and 3) technical challenges, including module failures, connectivity issues, and maintenance practices (Supplementary Data). Data collection utilised a combination of structured multiple-choice questions for standardised metrics (processing times, failure frequencies, facility characteristics) and open-ended questions to capture contextual factors and facility-specific challenges. The final section included space for additional comments and interviewer observations to ensure comprehensive data capture. For sample handling assessment, we specifically measured three critical time intervals: the time from sample arrival to processing initiation, the time from sample reagent (SR) addition to cartridge loading, and the time from cartridge loading to test initiation. These intervals were selected based on manufacturer recommendations and previous studies demonstrating their effect on test performance.^11,12^

Data collection was conducted through structured face-to-face interviews with laboratory managers and GeneXpert operators at each facility, using standardised questionnaires (Supplementary Data). Quantitative data were analysed using descriptive statistics to characterise patterns across facility types. The relationship between laboratory workload (categorised as 1–10, 10–25, 25–50, and >50 tests/day) and ordinal delay categories at each testing stage was assessed using Kruskal–Wallis tests (*P* < 0.05) using R software (v 4.5.1). Qualitative responses regarding challenges and potential solutions were systematically coded and categorised.

## RESULTS

The surveyed facilities included 49 hospitals and 4 health centres distributed across all five provinces, with inclusion of both urban and rural settings (Figure 1). The surveyed facilities demonstrated considerable heterogeneity in testing demand and operational context. The majority (94%) operated with a single GeneXpert machine, typically configured with four modules, regardless of facility size or patient volume. Small centres (those serving fewer than 50 patients daily) processed approximately 25–65 samples for TB weekly, while medium and large facilities managed substantially higher volumes, ranging from 50 to 120 TB samples weekly.

**Figure 2.**
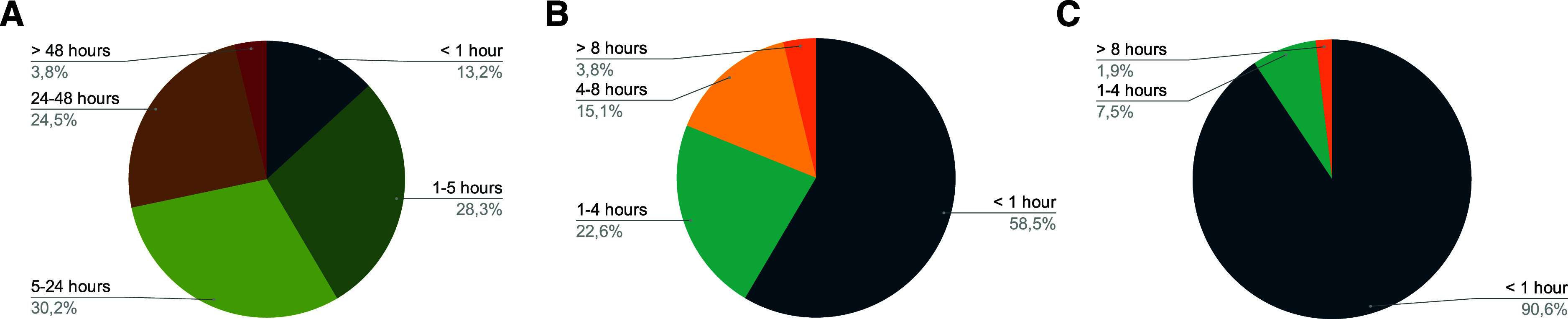
Overview of sample processing at the surveyed facilities, including **A:** time from sample arrival to processing; **B:** time from sample reagent addition to cartridge loading; and **C:** time to initiate GeneXpert testing after cartridge loading.

The timely processing of samples revealed considerable variation across the surveyed network (Figure 2). While 41% of facilities (22 sites) processed most samples within 5 h of receipt, the majority experienced some delays (Figure 2A). Nearly one third (30%, or 16 sites) reported routine delays of up to 24 h between sample receipt and processing initiation, and 25% (13 sites) experienced extended delays of 24–48 h. A small minority (4%, two sites) reflected that it took more than 48 h for the sample to begin processing. Facilities experiencing longer delays attributed these to receiving a high volume of samples, along with the need for additional machines to manage the workload, as they also process samples for HIV and hepatitis testing.

Regarding the cartridge loading after SR addition, 81% of facilities (43 sites) completed this step under a 4-h window (Figure 2B). Delays of 4–8 h were reported by 15% (eight sites), and 4% (two sites) answered that delays of more than 8 h might occur.

Test initiation after cartridge loading was the most consistently performed step, with 91% of facilities (48 sites) reporting immediate test initiation, typically within 1 h (Figure 2C). Four facilities (8%) reported routine delays of up to 4 h at this stage, and one site (2%) reported that delays might exceed 8 h. These delays were attributed to three primary factors: machine unavailability due to high testing volumes, staff shortages, and module failures requiring test redistribution to functioning modules.

The combined time from SR addition to test initiation is recommended to remain under 8 h. Among the surveyed sites, four facilities (8%) reported that delays across steps 2 and 3 could result in exceeding this 8-hour window. This occurred when cartridge loading delays of 4–8 h were followed by test initiation delays (n = 1), when cartridge loading itself exceeded 8 h (n = 2) or when test initiation exceeded 8 h (n = 1).

Kruskal–Wallis tests revealed no significant association between laboratory workload categories and delays at any of these processing stages: start to sample processing (step 1), *P* = 0.066; cartridge loading (step 2), *P* = 0.20, and test initiation (step 3), *P* = 0.69. The examination on whether delays in sample processing (step 1) were associated with subsequent delays in cartridge loading and test initiation (steps 2–3) showed no significant statistical associations (step 1 vs. step 2: *P* = 0.23; step 1 vs. step 3: *P* = 0.32). However, facilities with step 1 delays of 24–48 h showed more subsequent delays: only five sites (38%) completed step 2 within 1 h, three (23%) experienced 4–8 h delays, and two (15%) had delays exceeding 8 h. For step 3, 11 sites (85%) still completed this step within 1 h, though 1 facility (8%) reported delays exceeding 8 h.

Approximately 30% of sites (16 sites) explicitly reported insufficient GeneXpert capacity to meet their current testing demands. This shortage was especially pronounced in facilities serving dual diagnostic purposes, where competition between TB testing and other applications (particularly HIV viral load and hepatitis testing) created bottlenecks in the diagnostic workflow. Small and medium-sized facilities specifically emphasised the need for additional machines or an upgrade to devices with more than four modules to meet the demands of multiple diseases.

Beyond equipment limitations, 9% of facilities (five sites) expressed the need for additional staff to manage the workload. Additionally, five facilities advocated for the need for training, particularly focusing on machine maintenance and DataToCare connectivity troubleshooting, to improve operational efficiency.

Connectivity issues represented another significant technical barrier, with approximately 17% of sites (nine facilities) reporting internet reliability problems and/or problems with the DataToCare software platform. These connectivity challenges extended beyond mere reporting delays, as laboratory personnel described situations where DataToCare slowness caused delays in testing operations, likely due to workflow dependencies where staff wait for data entry before proceeding with subsequent tests.

Machine malfunctions emerged as a widespread challenge, with 81% of facilities (43 sites) reporting at least one GeneXpert module failure within the preceding year. While most (95%) described these failures as infrequent (occurring only once or twice per year), two facilities (5% of those reporting failures) experienced more frequent module malfunctions, with one site reporting persistent issues that occurred more than six times a year. Even occasional breakdowns had significant operational impacts, with 67% of affected facilities (29 out of 43) reporting that module failures directly caused delays in sample testing. In extreme cases, module failures led to complete testing interruptions, with one facility reporting a meaningful cessation of Xpert testing and another experiencing data loss due to machine breakdown. Two district hospitals identified high temperatures as a contributing factor to GeneXpert module failures.

The operational impact of these equipment failures was substantially amplified by maintenance support gaps. Seven facilities (13%) emphasised the need for preventive routine maintenance by Cepheid and a faster technical support process when modules fail, noting that prolonged downtimes lead to cartridge expiration.

## DISCUSSION

The operational challenges identified in this survey highlight important opportunities to enhance the efficiency of Rwanda’s GeneXpert platform usage. The substantial proportion of facilities experiencing delayed sample processing that exceeds 24 h is concerning from an operational efficiency perspective. However, it is essential to note that these delays remain within acceptable parameters for specimen stability. According to manufacturer guidelines, sputum sediments and unprocessed sputum can be stored at 2°C–8°C for up to 7 and 10 days, respectively, ensuring test performance is not compromised despite processing delays.

Interestingly, our analysis found no significant association between laboratory workload and processing delays at any testing stage (all *P* > 0.05), suggesting that operational challenges stem from facility-specific factors such as staffing levels, equipment availability, and resource management rather than testing volume alone (see Table). However, we observed a concerning pattern where facilities experiencing initial processing delays were more likely to encounter subsequent delays. This cascade effect suggests that early-stage delays create downstream operational pressure, potentially compromising test quality when combined delays exceed critical thresholds.

**Table.** Summary of key operational challenges, impact, and recommendations to improve GeneXpert usage.

Domain	Key findings	Impact on operations	Recommendations
Processing delays	59% of facilities experienced delays beyond 5 h in sample processing; 25% reported 24–48 h delays	Creates downstream operational pressure; facilities with a 24–48 h delay for step 1 showed a cascade effect (only 38% completed step 2 within 1 h)	Strategic machine allocation to facilities with consistent delays, considering the needs for TB and non-TB testing; advance notification when enhanced case finding are happening nearby a facility
Critical timing thresholds	8% of facilities (n = 4) reported potential to exceed the 8-h window from SR addition to test initiation	Risk of false-rifampicin-resistance due to prolonged SR exposure (>12 h not recommended by manufacturer)	Workflow optimisation training, with emphasis on time-sensitive processing steps
Machine capacity limitations	94% operated single four-module machines; 30% reported insufficient capacity; dual-purpose facilities faced TB/HIV/hepatitis testing competition	Bottlenecks in diagnostic workflow; delays in sample processing and test initiation	Additional machines for high-volume sites; upgrade to devices with >4 modules sample pooling strategies (up to four samples/cartridge), particularly for enhance/active case finding
Equipment failures	81% experienced at least one module failure in past year: 95% infrequent (1–2×/year), but 5% experienced >6 failures/year	Test redistribution to functioning modules; cartridge expiration during prolonged downtimes	Enhanced preventive maintenance schedules; faster technical support response; basic troubleshooting training for staff; environmental controls (air conditioning) where needed
Connectivity issues	17% reported internet reliability problems and DataToCare platform issues	Workflow disruptions when staff waits for data entry; delays beyond reporting; compromises real-time TB surveillance	Training on offline functionality; alternative connectivity options; web-based service tracking systems
Human resource gaps	9% requested additional staff; 9% identified training needs (machine maintenance, DataToCare troubleshooting)	Compounded delays during enhanced case finding; inability to resolve minor technical issues	Targeted staff support for high-burden facilities; longitudinal training cycles with performance feedback

SR = sample reagent.

Most delays were attributable to limited machine availability, which represent a significant opportunity for system optimisation through targeted interventions. Strategic machine allocation should be prioritised, with additional GeneXpert capacity directed to high-volume facilities consistently reporting processing delays. This approach is supported by the DNO analyses demonstrating that strategic placement decisions should consider maximising utilisation of existing capacity.^7^ Sample pooling, where up to four sputum samples are processed in a single cartridge, represents another promising approach to address high-volume demands if the positivity rate is relatively low. This strategy has demonstrated maintained sensitivity while reducing cartridge costs in several settings.^13,14^ Pooling could simultaneously optimise machine utilisation and reduce per-test costs, though implementation would require validation in Rwanda’s specific epidemiological context.

For high-volume facilities serving dual diagnostic purposes, physically separating TB and non-TB testing workflows through dedicated machines would reduce competition for limited resources and streamline operations, provided that each machine meets minimum daily throughput requirements for cost-effective utilisation. Some facilities also requested advance notice preceding the initiation of enhanced TB case finding to allow better planning and preparedness. Particularly those health facilities located near correctional facilities or documented TB hotspots may need additional staff support to manage the workload effectively during enhanced case finding.

The finding that four facilities report occasional delays of more than 8 h when loading the cartridge after SR addition warrants further investigation. Prolonged incubation in SR has been shown to affect the specificity of rifampicin-resistance detection in paucibacillary samples, attributed to C-to-T or G-to-A substitutions from extended exposure to NaOH, a component of the SR.^12^ Hence, prolonged SR incubation beyond 12 h is not recommended by the manufacturer, although other studies suggest that incubation up to 24 h may be acceptable without significant impact on test performance.^12^ Notably, neither of these two facilities contributed to the false-rifampicin-resistant results reported in the recent Ultra analysis.^9^ This practice may reflect knowledge gaps among laboratory staff regarding the optimal timing for SR addition, as staff would likely delay adding SR until functional slots become available if they understood the risks associated with extended incubation times.

At least one module failure was reported by 81% of facilities in the last year, representing a substantial vulnerability in the diagnostic network when viewed at the system level. Equipment maintenance and servicing remain a challenge despite improvements following the NTP’s adoption of the manufacturer’s maintenance approach. These issues are a notorious challenge that has been previously studied in several settings, showing problems such as high levels of module failure and unavailability of spare modules.^3^ The current system includes scheduled yearly preventive maintenance visits (or after 2,000 sample volumes) as part of the Cepheid contract, along with machine calibration procedures. However, the prolonged response times for technical support following module failures, which rely on manufacturer technicians, indicate a critical need for more responsive maintenance algorithms. While facilities can perform routine preventive maintenance tasks, they cannot replace modules or conduct curative maintenance without Cepheid certification, creating dependencies on external technical support for equipment failures. The prolonged response times for technical support following module failures indicate opportunities for improvement in the current approach. Expanding basic troubleshooting training for facility staff could enable resolution of minor issues without external intervention, though major repairs would still require certified technicians. Additionally, enhanced service tracking systems, such as web-based monitoring of instrument maintenance status, could improve oversight and response coordination.

Similarly, the connectivity challenges affecting 17% of facilities highlight how digital health infrastructure limitations can undermine even well-designed laboratory networks, resulting in geographic disparities in diagnostic efficiency. Connectivity solutions should be developed to address the reliability issues affecting the DataToCare system, which is not only necessary for the functioning of health facilities but also crucial for ensuring a robust nationwide data collection that enables real-time surveillance of evolving TB drug resistance. While DataToCare is designed to function offline with automatic data synchronisation upon connectivity restoration, the reported workflow disruptions suggest implementation challenges rather than technical limitations. Enhanced training on utilising these existing offline capabilities and alternative connectivity options would enable staff to maintain operations during internet outages without workflow interruptions, thereby improving the acceptance of the software.

The training gaps identified among 9% of facilities, particularly regarding machine maintenance and DataToCare troubleshooting, reflect broader implementation challenges documented globally for molecular diagnostic platforms. Systematic reviews emphasise that high-quality, regular, longitudinal education and training with performance feedback for all health workers is critical to strengthen mWRD implementation and maximise diagnostic efficiency.^6^

Implementing environmental controls through air conditioning systems represents an often-overlooked opportunity for system optimisation. Two district hospitals specifically identified high temperatures as contributing factors to module failures, consistent with manufacturer specifications regarding operating conditions.^4^ Where necessary, the installation of air conditioning in GeneXpert rooms would represent a cost-effective investment to protect the more substantial investment in the diagnostic technology itself.

This study has several notable strengths, including comprehensive geographical coverage across all five provinces of Rwanda and representation of facilities with varying patient volumes and operational contexts. However, some limitations should be acknowledged. The sampling of 53 facilities, due to logistical constraints, may not fully capture the operational experiences of all 73 GeneXpert sites nationwide. Besides, self-reported data may be subject to recall bias, particularly regarding historical equipment failures and processing times. Finally, this study focuses on GeneXpert operations from the sample reception at equipped facilities. The potential pre-analytical factors like specimen collection and transportation, while important for diagnostic quality, were outside the scope of this analysis.

## CONCLUSION

This assessment of Rwanda’s GeneXpert experiences reveals critical operational challenges that limit the full potential of this advanced diagnostic technology. The issues identified are not unique to Rwanda but reflect the broader difficulties of implementing advanced diagnostic technologies in resource-constrained settings.^3^ Our findings highlight several key areas for intervention: strategic machine allocation, increased staff training, enhanced connectivity solutions, improved maintenance protocols, and implementation of environmental controls. Our findings support the growing evidence that successful mWRD implementation requires multicomponent strategies that optimise clinical and laboratory processes while addressing health system strengthening needs.^6^ These recommendations align with WHO guidance and represent a comprehensive approach to addressing the multifaceted challenges identified. As molecular diagnostic platforms expand throughout resource-limited settings, addressing these operational inefficiencies becomes increasingly important. Through systematic improvements, Rwanda can enhance its TB diagnostic capacity and potentially serve as a model for effective molecular diagnostic implementation for other diseases and in similar settings.

## Supplementary Material

## References

[1] Helb D, Rapid detection of Mycobacterium tuberculosis and rifampin resistance by use of on-demand, near-patient technology. J Clin Microbiol. 2010;48(1):229-237. 19864480 10.1128/JCM.01463-09 PMC2812290

[2] Chakravorty S, The new Xpert MTB/RIF ultra: improving detection of Mycobacterium tuberculosis and resistance to rifampin in an assay suitable for point-of-care testing. mBio. 2017;8(4):e00812-17. 28851844 10.1128/mBio.00812-17 PMC5574709

[3] Albert H, Development, roll-out and impact of Xpert MTB/RIF for tuberculosis: what lessons have we learnt and how can we do better? Eur Respir J. 2016;48(2):516-525. 27418550 10.1183/13993003.00543-2016 PMC4967565

[4] World Health Organization. Xpert MTB/RIF implementation manual: technical and operational ‘how-to’; practical considerations. Geneva: WHO, 2014. 25473699

[5] Hanrahan CF, Implementation of Xpert MTB/RIF in Uganda: missed opportunities to improve diagnosis of tuberculosis. Open Forum Infect Dis. 2016;3(2):ofw068. 27186589 10.1093/ofid/ofw068 PMC4866550

[6] Nathavitharana RR, Implementation strategies to increase the uptake and impact of molecular WHO-recommended rapid diagnostic tests: evidence from a mixed-methods systematic review. BMJ Glob Health. 2025;10(9):e018700. 10.1136/bmjgh-2024-018700 PMC12458786 40967658

[7] Albert H, Optimizing diagnostic networks to increase patient access to TB diagnostic services: development of the diagnostic network optimization (DNO) approach and learnings from its application in Kenya, India and the Philippines. PLoS One. 2023;18(11):e0279677. 38033120 10.1371/journal.pone.0279677 PMC10688908

[8] Rwanda Biomedical Centre. Tuberculosis and other respiratory communicable diseases division annual report 2020-2021. Rwanda Biomedical Centre, 2021. https://www.ccm.rw/fileadmin/user_upload/Annual%20report%20TB%20%20ORD%202020%202021.pdf.

[9] Cuella-Martin I, Paucibacillary tuberculosis drives the low positive predictive value of Xpert MTB/RIF ultra for rifampicin resistance detection in low-prevalence settings. Clin Infect Dis. 2025;22:ciaf132. 10.1093/cid/ciaf132 PMC12448619 40120087

[10] Rwanda Biomedical Centre. Tuberculosis and other respiratory communicable diseases division annual report 2023-2024. Rwanda Biomedical Centre, 2024. https://www.rbc.gov.rw/fileadmin/user_upload/report_2024/TB/TBORD_annual_report_2023-2024.pdf.

[11] Cepheid. Xpert MTB/RIF ultra. Instructions for use. Cepheid, 2023. https://infomine.cepheid.com/sites/default/files/2024-01/302-5776%20Rev.%20D%20IFU%20MTB.RIF%20Ultra%20ENGLISH%20MII.pdf.

[12] Banada PP, Containment of bioaerosol infection risk by the Xpert MTB/RIF assay and its applicability to point-of-care settings. J Clin Microbiol. 2010;48(10):3551-3557. 20720033 10.1128/JCM.01053-10 PMC2953088

[13] Chry M, Can the high sensitivity of Xpert MTB/RIF ultra be harnessed to save cartridge costs? Results from a pooled sputum evaluation in Cambodia. Trop Med Infect Dis. 2020;5(1):27. 32075250 10.3390/tropicalmed5010027 PMC7157618

[14] Cuevas LE, Systematic review of pooling sputum as an efficient method for Xpert MTB/RIF tuberculosis testing during the COVID-19 pandemic. Emerg Infect Dis. 2021;27(3):719-727. 33622482 10.3201/eid2703.204090 PMC7920689

