# Supplementary material for: Key operational challenges at a programmatic level to achieve optimal use of the GeneXpert platform
Source: IJTLD Open. 2026 May 11;3(5):298–304. doi: 10.5588/ijtldopen.25.0630 (PMC13160268; doi:10.5588/ijtldopen.25.0630)
Supplement: Supplementary file 1 [file ijtldopen25-0630_supplementarydata1.pdf]

Supplementary material

Questionnaire: GeneXpert Machine Use and Sample Processing

Facility Details

1. **Name of Health Facility:____________**
2. **Location (City/Town/Region):**
3. **Size of Facility:**
    - o Small (1-50 patients per day)
    - o Medium (51-200 patients per day)
    - o Large (201+ patients per day)

GeneXpert Machine Usage

4. **How many GeneXpert machines do you have at your facility?**
    - o 1
    - o 2-3
    - o More than 3
5. **How often do you use the GeneXpert machine?**
    - o Daily
    - o Weekly
    - o Bi-weekly
    - o Monthly
6. **How many tests do you process on the GeneXpert machine?**
    - o Per day: ____________
    - o Per week: ______________

Sample Processing and Handling

7. **How long does it take for a sample shipped to the facility to be processed by the GeneXpert machine?**
    - o Less than 1 hour
    - o 1-5 hours
    - o 5-24 hours
    - o 24-48 hours
    - o More than 48 hours
8. **Once the sample reagent (SR) is added to the specimen, how long might it be delayed before loading to the cartridge?**
    - o Less than 1 hour
    - o 1-4 hours
    - o 4-8 hours
    - o More than 8 hours
9. **Once a sample is loaded into the GeneXpert cartridge, how long might it be delayed before the machine starts processing it?**
    - o Less than 1 hour
    - o 1-4 hours
    - o 4-8 hours
    - o More than 8 hours
10. **Have you experienced any issues with the GeneXpert machine delaying sample processing?**
    - o Yes
    - o No
11. **If yes, what were the main reasons for the delay?**
    - o Too many samples waiting
    - o Broken machine/module
    - o Lack of staff
    - o Other (please specify): N/A

Machine Issues

12. **Have you experienced broken modules on your GeneXpert machine in the past year?**
    - o Yes
    - o No

13. **If yes, how often do these issues occur?**
    - Rarely (1-2 times a year)
    - Occasionally (3-6 times a year)
    - Frequently (more than 6 times a year)
14. **How do broken modules impact your ability to process samples?**
    - No significant impact
    - Delays testing of samples in general
    - Delays loading of cartridges after adding the SR buffer
    - Delays loading of filled cartridges in the machine
    - Causes cartridges to expire
    - Other (please specify): ______________

Comments

15. **Do you have any other comments or suggestions on how to improve GeneXpert machine operations or sample handling at your facility?**

    ____

Thank you for taking the time to complete this survey. Your input is valuable to improving the use and maintenance of GeneXpert machines.
